# Effect of riboflavin deficiency on development of the cerebral cortex in *Slc52a3* knockout mice

**DOI:** 10.1038/s41598-020-75601-9

**Published:** 2020-10-28

**Authors:** Congyun Jin, Atsushi Yonezawa, Hiroki Yoshimatsu, Satoshi Imai, Madoka Koyanagi, Kaori Yamanishi, Shunsaku Nakagawa, Kotaro Itohara, Tomohiro Omura, Takayuki Nakagawa, Junya Nagai, Kazuo Matsubara

**Affiliations:** 1 Department of Clinical Pharmacology and Therapeutics, Kyoto University Hospital, 54 Shogoin Kawahara-cho, Sakyo-ku, Kyoto, 606-8507 Japan; 2 Graduate School of Pharmaceutical Sciences, Kyoto University, 54 Shogoin Kawahara-cho, Sakyo-ku, Kyoto, 606-8507 Japan; 3 Department of Pharmaceutics, Osaka University of Pharmaceutical Sciences, 4-20-1 Nasahara, Takatsuki, Osaka 569-1094 Japan

**Keywords:** Neurochemistry, Experimental models of disease, Malnutrition

## Abstract

Riboflavin transporter 3 (RFVT3), encoded by the *SLC52A3* gene, is important for riboflavin homeostasis in the small intestine, kidney, and placenta. Our previous study demonstrated that *Slc52a3* knockout (*Slc52a3*−/−) mice exhibited neonatal lethality and metabolic disorder due to riboflavin deficiency. Here, we investigated the influence of *Slc52a3* gene disruption on brain development using *Slc52a3*−/− embryos. *Slc52a3*−/− mice at postnatal day 0 showed hypoplasia of the brain and reduced thickness of cortical layers. At embryonic day 13.5, the formation of Tuj1^+^ neurons and Tbr2^+^ intermediate neural progenitors was significantly decreased; no significant difference was observed in the total number and proliferative rate of Pax6^+^ radial glia. Importantly, the hypoplastic phenotype was rescued upon riboflavin supplementation. Thus, it can be concluded that RFVT3 contributes to riboflavin homeostasis in embryos and that riboflavin itself is required during embryonic development of the cerebral cortex in mice.

## Introduction

Riboflavin (vitamin B2) is essential for cellular growth and function. It is enzymatically converted to flavin mononucleotide (FMN) and flavin adenine dinucleotide (FAD), which participate in metabolic oxidation–reduction reactions of carbohydrates, amino acids, and lipids^1^. Riboflavin deficiency leads to a variety of clinical abnormalities, such as cataracts, skin disorders, anemia, growth retardation, migraine, and peripheral neuropathy^2–4^. In addition, riboflavin-deficient rat models have indicated that vitamin B2 is indispensable during early postnatal development of the brain^5^. Riboflavin transporters (RFVT1–3) play an important role in the absorption and tissue distribution of water-soluble riboflavin, and are essential for maintaining its homeostasis^6–9^.

RFVT3, encoded by the *SLC52A3* gene, consists of 469 amino acids with 11 putative membrane-spanning domains^7,9^. RFVT3 mRNA is highly expressed in the testis, small intestine, kidney, and placenta, but rarely in the brain^7,9,10^. Riboflavin, but not FMN or FAD, is transported by RFVT3^10,11^. In a previous study, we investigated the physiological role of RFVT3 using *Slc52a3* knockout (*Slc52a3*−/−) mice. *Slc52a3*−/− mice exhibited neonatal lethality, with hyperlipidemia and hypoglycemia, owing to riboflavin deficiency^12^. Recently, mutations in the *SLC52A3* gene have been documented in patients with a neurological disorder known as Brown-Vialetto-Van Laere (BVVL) syndrome^13–15^. Low plasma riboflavin levels, abnormal fatty acid metabolism, and neurological symptoms have been observed in patients with RFVT3 mutations; however, these features could be rescued by supplementation with riboflavin^16–27^. Human induced pluripotent stem cell-derived motor neurons from BVVL syndrome patients with *SLC52A3* mutations were generated to explore the effect of riboflavin transporter mutations^28^. This in vitro disease model indicated a reduction in axonal growth and altered cytoskeletal functionality. However, the relationship between *SLC52A3* gene disruption and neurological pathophysiology remains unclear.Here, we investigated the role of riboflavin in brain development using *Slc52a3*−/− mice.

## Results

### Histological analysis of postnatal day 0 (P0) mice brain

**Figure 1:**
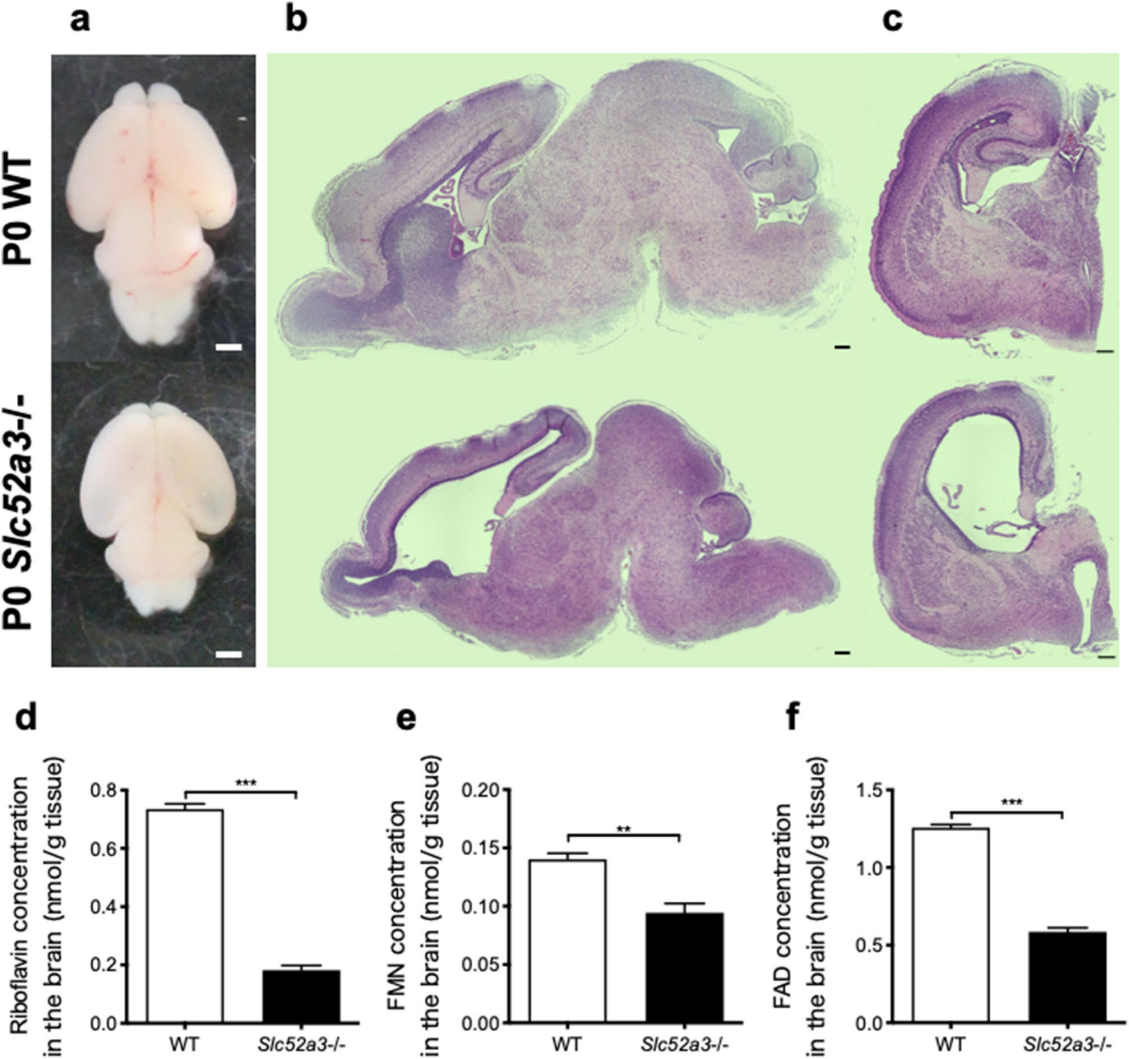
Abnormal brain formation in P0 *Slc52a3*−/− mice. (**a**) Whole-mount brains. Scale bars, 1 mm. (**b**,**c**) H&E-stained sagittal and coronal sections from the indicated genotypes. Scale bars, 200 μm. (**d**–**f**) Riboflavin, FMN, and FAD concentrations in P0 brains from WT (n = 3 from 1 dam) and *Slc52a3*−/− (n = 4 from 2 dams) mice. Each bar represents the mean ± S.E.M., **P < 0.01, ***P < 0.001, WT vs *Slc52a3*−/−.

We have previously reported that organogenesis of newborn *Slc52a3*−/− mice appeared to be normal, although their body weights were significantly lower than those of wild-type (WT) mice^12^. Macroscopically, the *Slc52a3*−/− brain showed increased transparency in the dorsal telencephalon compared with the WT brain (Fig. 1a). Histological analysis of the *Slc52a3*−/− brain revealed hypoplasia and global developmental delay. The *Slc52a3*−/− brain had enlarged lateral ventricles but particularly smaller cerebral cortex, striatum, hippocampus, and cerebellum (Fig. 1b,c). Map2^+^ neurons were clearly detected in *Slc52a3*−/− brains, accompanied by a reduction in astrocytes and an increase in microglia in the cerebral cortex (Supplementary Fig. 1). Brain riboflavin, FMN, and FAD levels were significantly lower in *Slc52a3*−/− mice compared with WT animals (Fig. 1d–f). In situ hybridization and real-time PCR (RT-PCR) analysis indicated that *Slc52a3* transcripts were absent or rare in the brain (Supplementary Fig. 2).

### Cortical layer formation in P0 Slc52a3−/− mice

**Figure 2:**
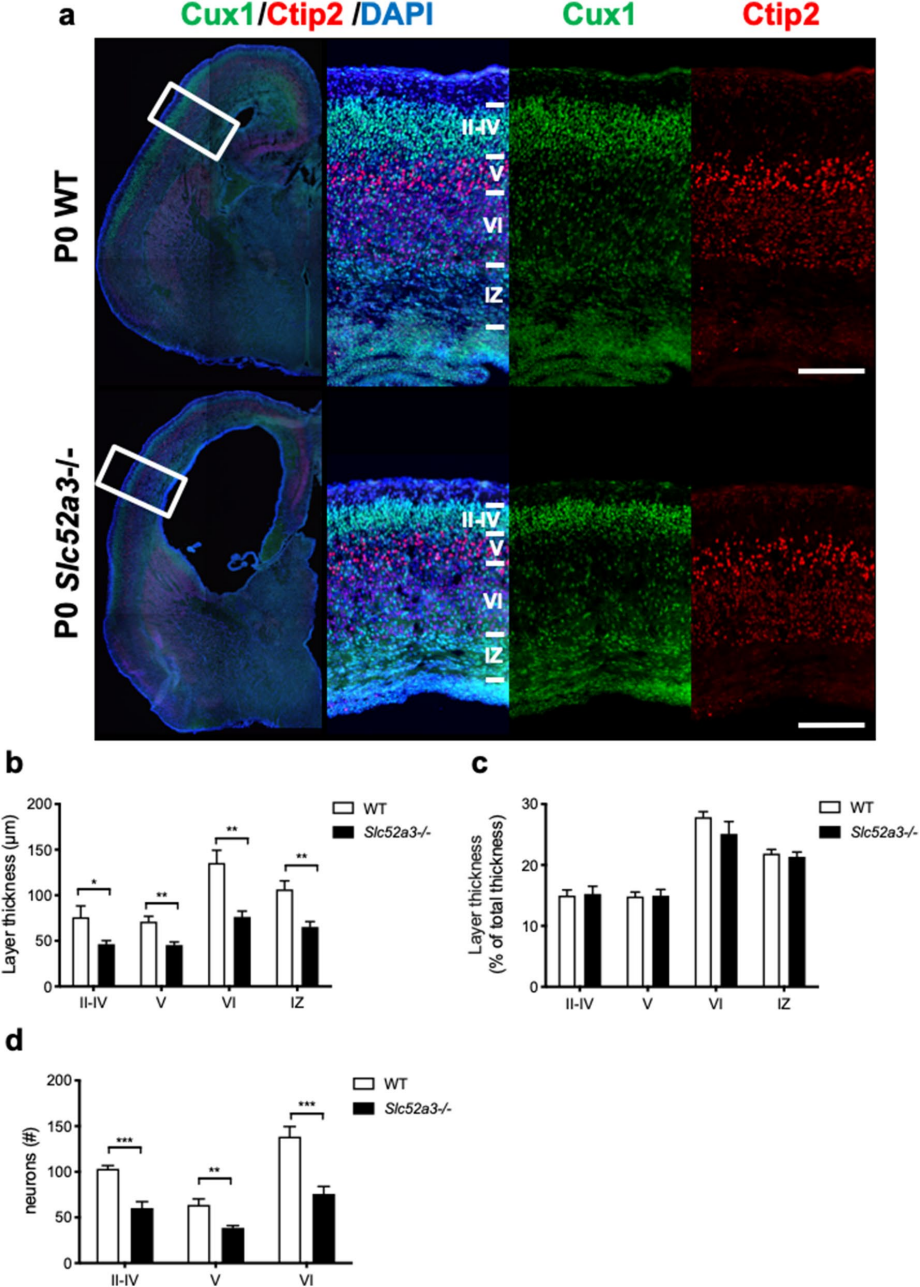
Cortical layer formation in P0 WT and *Slc52a3*−/− mice. (**a**) Coronal sections stained with DAPI and antibodies against Cux1 (green, highly expressed in layers II–IV) and Ctip2 (red, highly expressed in layer V and weakly expressed in layer VI). Boxed areas indicate magnified regions in the right panels. Scale bars, 100 μm. (**b**) Thickness of each layer and intermediate zone. (**c**) Thickness of each cortical layer and intermediate zone relative to total cortical thickness expressed as a percentage. n = 7 from 7 dams. (**d**) Graph representing the number of Cux1^+^ and Ctip2^+^ cells within 150-μm column (WT, n = 5; *Slc52a3*−/−, n = 7). Each bar represents the mean ± S.E.M., *P < 0.05, **P < 0.01, WT vs. *Slc52a3*−/−. IZ: intermediate zone.

To assess cortical lamination in *Slc52a3*−/− mice, we stained brain sections with anti-Cux1 antibody, which identified layers II to IV, and anti-Ctip2 antibody, which labeled strongly layer V and weakly layer VI (Fig. 2a). All cortical layers as well as the intermediate zone (Fig. 2b) were markedly thinner in *Slc52a3*−/− brains, but revealed no difference in laminar formation when compared with the total cortical thickness (Fig. 2c). *Slc52a3*−/− P0 brains also contained fewer Cux1^+^ and Ctip2^+^ neurons (Fig. 2d). The terminal deoxynucleotidyl transferase-mediated deoxyuridine triphosphate-digoxigenin nick end labeling (TUNEL) assay indicated no obvious changes in cell apoptosis in *Slc52a3*−/− brains (Supplementary Fig. 3).

### Neurogenesis in embryonic day 13.5 (E13.5) Slc52a3−/− embryos

**Figure 3:**
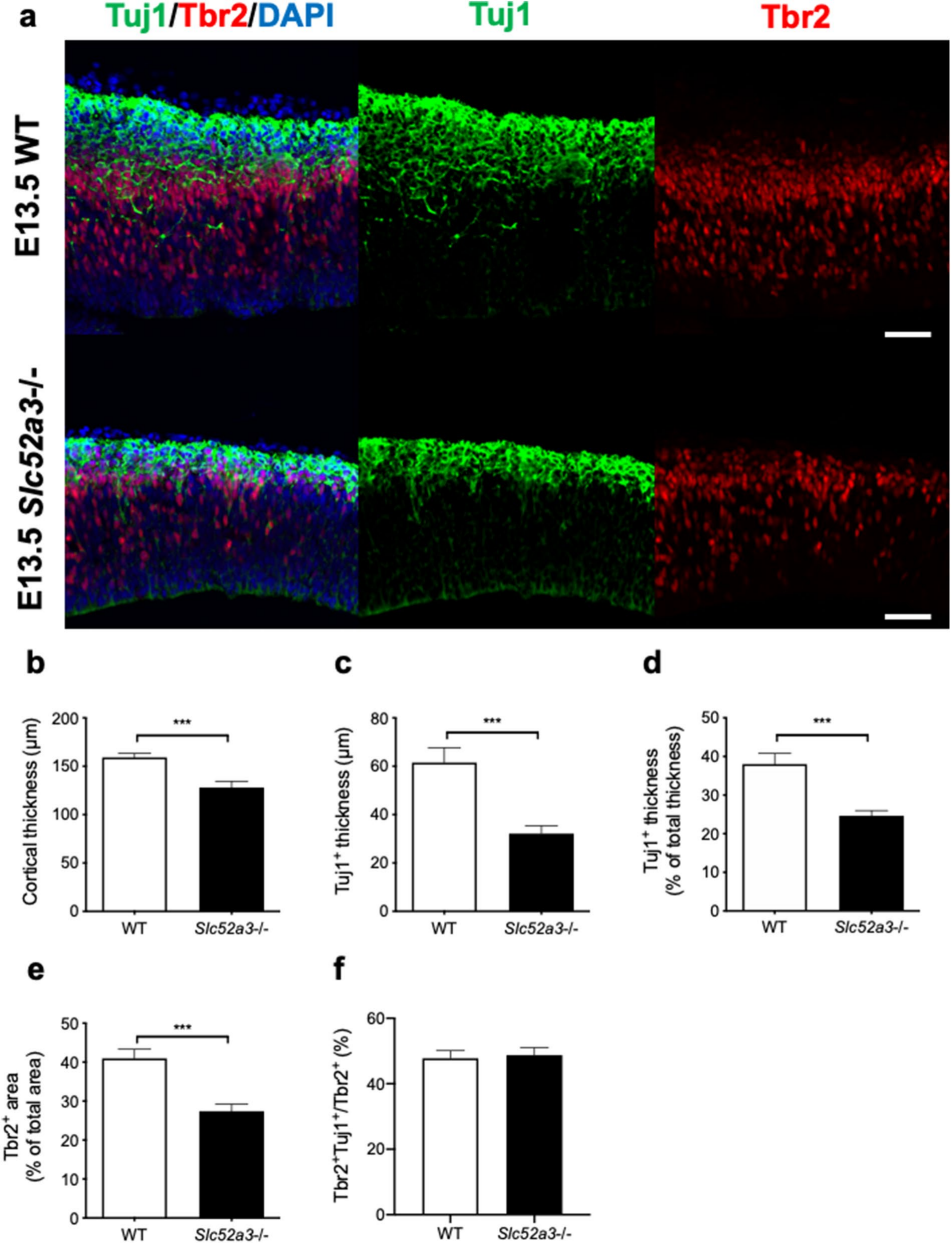
Neurogenesis in E13.5 WT and *Slc52a3*−/− embryos. (**a**) Coronal sections stained with DAPI and antibodies against Tuj1 (green, immature neurons) and Tbr2 (red, intermediate neural progenitors). Scale bars, 50 μm. (**b**) Thickness of the total cortex. (**c**) Thickness of the Tuj1^+^ layer. (**d**) Thickness of the Tuj1^+^ layer relative to total cortical thickness and expressed as a percentage. (**e**) Percentage of the Tbr2^+^ area relative to the DAPI^+^ area of the cortex. n = 11 from 9 dams. (f) Percentage of Tbr2^+^ cells that are Tuj1^+^. Each bar represents the mean ± S.E.M., ***P < 0.001, WT vs. *Slc52a3*−/−.

Next, we examined cortical development in E13.5 embryos. Immature neurons and intermediate neural progenitors were stained with antibodies against Tuj1 and Tbr2, respectively (Fig. 3a). *Slc52a3*−/− brains showed reduced thickness of the total cortex (Fig. 3b), the Tuj1^+^ layer (Fig. 3c,d), and the extent of the Tbr2^+^ area relative to the total cortex (Fig. 3e). Tuj1^+^ neurons and Tbr2^+^ intermediate neural progenitors were markedly fewer in the cerebral cortex of *Slc52a3*−/− embryos (Supplementary Fig. 4a,b), along with a reduction in *Tbr2* mRNA levels (Supplementary Fig. 4c). The ratio of Tbr2^+^Tuj1^+^ cells to Tbr2^+^ cells in WT and *Slc52a3−/−* brains was similar (Fig. 3f). Brain sections from *Slc52a3*−/− embryos were TUNEL-negative (Supplementary Fig. 3).

### Cell proliferation of radial glia in E13.5 Slc52a3−/− embryos

**Figure 4:**
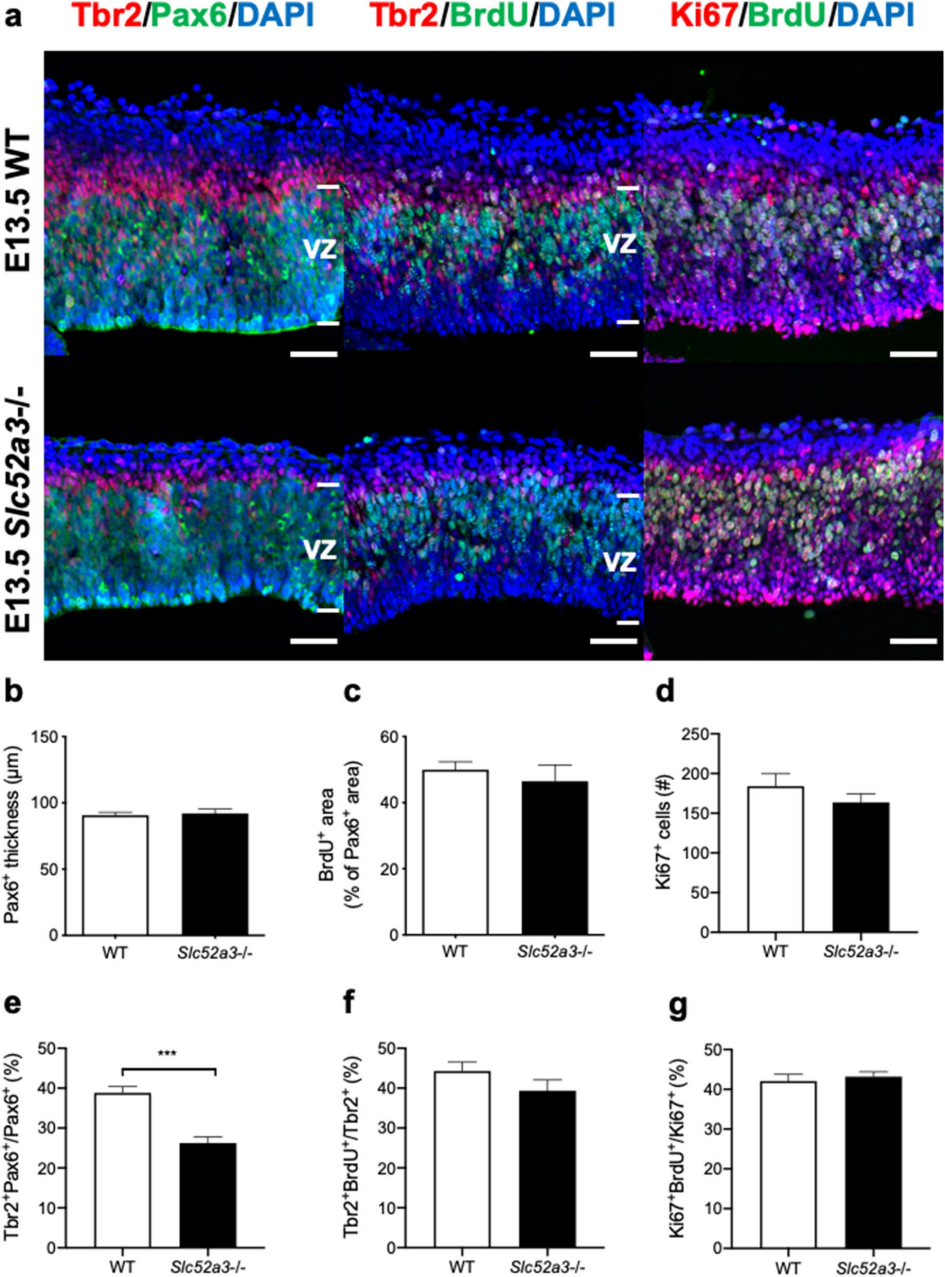
Cell proliferation of radial glia in embryonic cerebral cortex at E13.5 in WT and *Slc52a3*−/− mice. (**a**) Coronal sections stained with DAPI and antibodies against Tbr2 (red, intermediate neural progenitors), Pax6 (green, radial glia in the ventricular zone), BrdU (green, proliferating cells) and Ki67 (red, proliferating cells). Pregnant females were dissected 1 h after BrdU injection. Scale bars, 50 μm. (**b**) Thickness of the Pax6^+^ ventricular zone. (**c**) Percentage of the BrdU^+^ area relative to the DAPI^+^ area in the ventricular zone. (**d**) Number of Ki67^+^ cells per 85-μm column. (**e**) Percentage of Pax6^+^ cells that are Tbr2^+^. (**f**) Percentage of Tbr2^+^ cells that are BrdU^+^. (**g**) Percentage of Ki67^+^ cells that are BrdU^+^. n = 3–11 from 3 to 9 dams. Each bar represents the mean ± S.E.M., ***P < 0.001, WT vs. *Slc52a3*−/−. VZ: ventricular zone.

Radial glia in the ventricular zone were stained with antibodies against Pax6 and cell proliferation was determined by bromodeoxyuridine (BrdU) and Ki67 detection (Fig. 4a). No difference in thickness of the ventricular zone (Fig. 4b) or number of Pax6^+^ radial glia (Supplementary Fig. 4d) was observed between WT and *Slc52a3*−/− embryos, which was supported by RT-PCR analyses (Supplementary Fig. 4e). Similarly, no difference between WT and *Slc52a3*−/− embryos was noted regarding the extent of the BrdU^+^ area in the ventricular zone (Fig. 4c). The number of Ki67^+^ cells was similar in WT and *Slc52a3*−/− embryos (Fig. 4d). Pax6/Tbr2 double immunofluorescence revealed that the ratio of Pax6^+^ cells expressing Tbr2 was significantly lower in *Slc52a3*−/− embryos (Fig. 4e). In contrast, no statistically significant difference was found in the ratios of Tbr2^+^BrdU^+^ to Tbr2^+^ cells (Fig. 4f) and Ki67^+^BrdU^+^ to Ki67^+^ cells (Fig. 4g) between WT and *Slc52a3*−/− embryos.

### Supplementation of riboflavin in Slc52a3−/− mice

**Figure 5:**
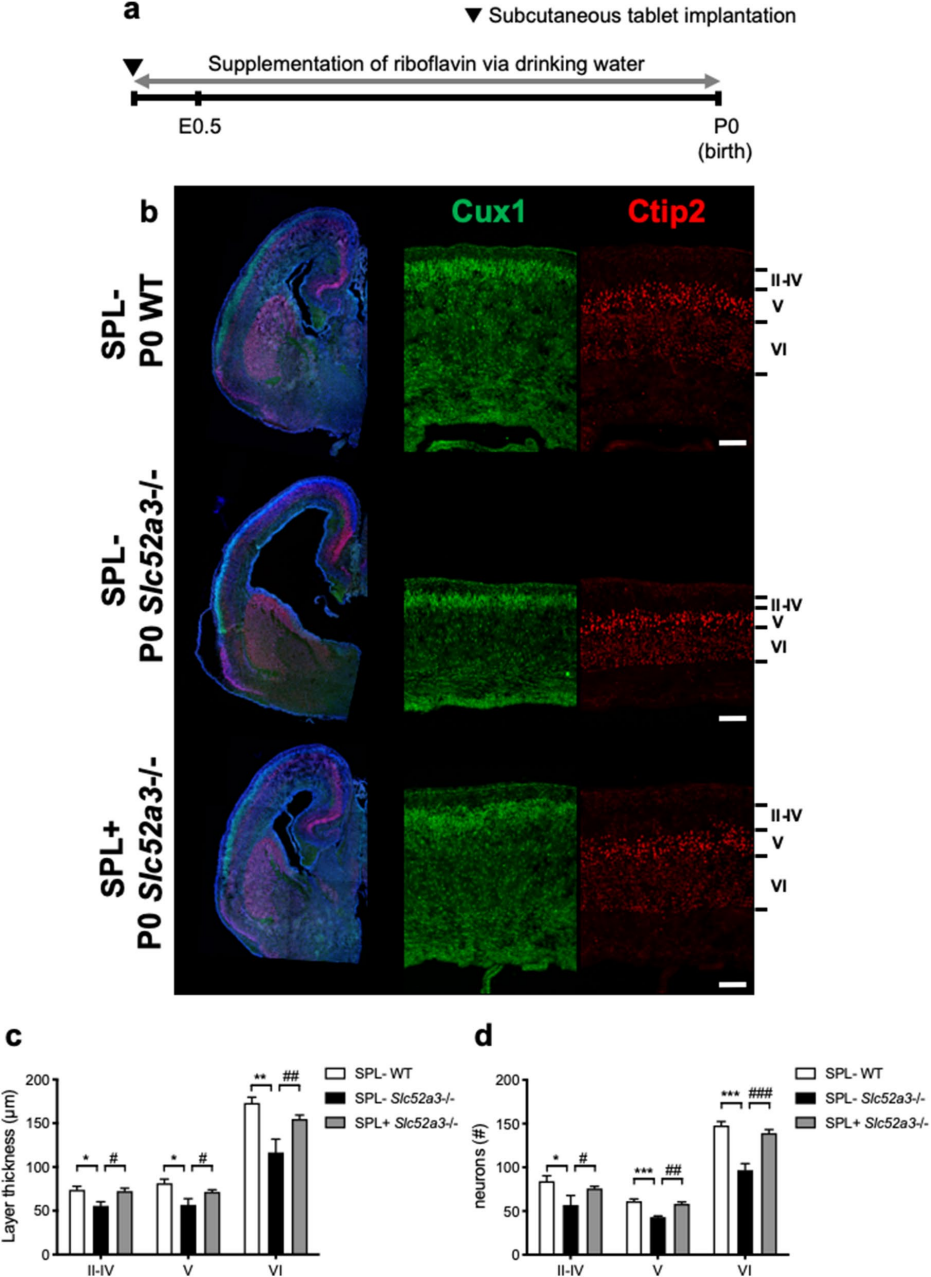
Supplementation of riboflavin in *Slc52a3*−/− mice. (**a**) Protocol for riboflavin supplementation (SPL+). *Slc52a3*+/− female mice were supplemented with a 5 mg riboflavin tablet by subcutaneous implantation and with 50 mg/L riboflavin in drinking water supplied ad libitum from the onset of crossbreeding. The negative control group (SPL−) was given a placebo tablet and drinking water without riboflavin. (**b**) Coronal sections from WT and *Slc52a3*−/− mice from the SPL+ and SPL− groups stained with antibodies against Cux1 and Ctip2. Scale bars, 100 μm. (**c**) Thickness of cortical brain layers in SPL− WT (n = 6 from 2 dams), SPL− *Slc52a3*−/− (n = 5 from 3 dams), and SPL+ *Slc52a3*−/− (n = 20 from 8 dams) mice. (**d**) Number of Cux1^+^ and Ctip2^+^ cells per 150-μm column (SPL− WT, n = 6; SPL− *Slc52a3*−/−, n = 4; SPL+ *Slc52a3*−/−, n = 18). Each bar represents the mean ± S.E.M., *P < 0.05, **P < 0.01, ***P < 0.001, SPL− WT vs. SPL− *Slc52a3*−/−; ^#^P < 0.05, ^##^P < 0.01, ^###^P < 0.001, SPL− *Slc52a3*−/− vs. SPL+ *Slc52a3*−/−.

*Slc52a3*+/−  female mice were supplemented by subcutaneous implantation of a 5 mg riboflavin tablet and ad libitum administration of 50 mg/L of riboflavin in drinking water from gestational day 0 (Fig. 5a). The body weights of *Slc52a3*−/− mice without riboflavin supplementation (1.15 ± 0.02 g, n = 5) were significantly lower than those of WT mice (1.38 ± 0.03 g, n = 6, p < 0.001) but rescued with riboflavin supplementation (1.32 ± 0.03 g, n = 20, p = 0.003, vs. *Slc52a3*−/− without riboflavin supplementation). Examination of cerebral cortex development in P0 *Slc52a3*−/− mice with or without riboflavin supplementation revealed that riboflavin addition rescued the appearance of the lateral ventricles, cerebral cortex, striatum, and hippocampus (Fig. 5b). Moreover, it significantly increased the thickness of cortical layers, the cell number of layer neurons in *Slc52a3*−/− mice (Fig. 5c,d), and *Tbr2* mRNA levels (Supplementary Fig. 4c).

## Discussion

In the present study, we investigated the role of riboflavin in cerebral cortex development in mice. *Slc52a3*−/− mice exhibited abnormal brain morphology as a consequence of lower riboflavin levels. Histological analysis of the cerebral cortex showed decreased thickness of cortical layers at P0 and E13.5. *Slc52a3*−/− embryos displayed fewer immature neurons and intermediate neural progenitors in the cerebral cortex, and none of them showed signs of cell death. Importantly, brain hypoplasia was rescued by riboflavin supplementation. Taken together, these findings indicate that RFVT3 contributes to riboflavin homeostasis in embryos and plays an important role during embryonic development of the cerebral cortex in mice.

**Figure 6:**
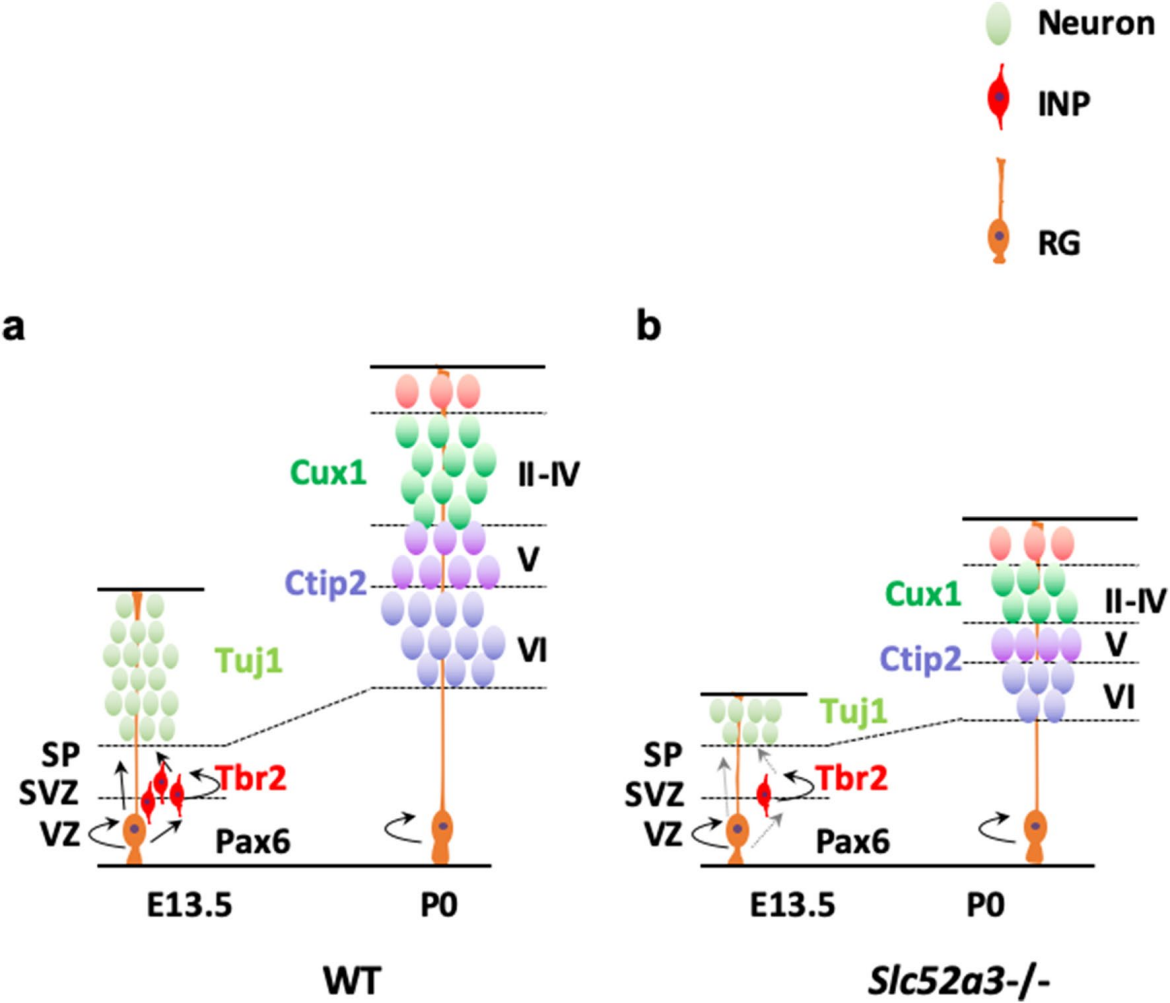
Models depicting the development of the cerebral cortex. (**a**) Pax6^+^ radial glia in the ventricular zone produce Tbr2^+^ intermediate neural progenitors and Tuj1^+^ immature neurons at E13.5. Then, Tuj1^+^ immature neurons migrate along radial glia to their final laminar destinations during cortical neurogenesis. Finally, brains show a six-layered cerebral cortex at P0. (**b**) *Slc52a3*−/− mice produce fewer intermediate neural progenitors and neurons resulting in atrophy of the cerebral cortex. SP: subplate; SVZ: subventricular zone; VZ: ventricular zone; INP: intermediate neural progenitor; RG: radial glia.

The six-layered mammalian cerebral cortex is formed through the coordinated processes of neurogenesis and migration (Fig. 6a)^29^. During cortical neurogenesis, radial glia undergo proliferative divisions aimed at self-renewal and neurogenic divisions to generate either neurons or intermediate neural progenitors. Progenitors in the ventricular and subventricular zones divide and give rise to neurons specific to each layer. Early-born neurons populate layers VI and V, whereas late-born neurons migrate past them to progressively populate layers IV and II/IV. Immunostaining of P0 *Slc52a3*−/− brain sections showed that the thickness of each cortical layer, including the intermediate zone, and the number of layer neurons were significantly decreased in absolute but not in relative terms. Disruption of the *Slc52a3* gene was associated with a reduction in the number of neurons in every cortical layer, instead of just a specific one. At E13.5, Tbr2^+^ intermediate neural progenitors and Tuj1^+^ neurons were significantly fewer but without signs of cell death. In contrast, there was no change in the population of Pax6^+^ radial glia and BrdU^+^ cell proliferation. Pax6/Tbr2 double immunofluorescence revealed that the proportion of Pax6^+^ cells expressing Tbr2 was significantly lower in *Slc52a3*−/− embryos. However, the proportion of Tbr2^+^BrdU^+^ and Tbr2^+^Tuj1^+^ cells were similar in WT and *Slc52a3*−/− embryos. These results suggest that the transition from intermediate neural progenitors to neurons and the proliferation of intermediate neural progenitors were similar in WT and *Slc52a3*−/− embryos; however, differentiation from radial glia to intermediate neural progenitors decreased in *Slc52a3*−/− embryos. The sequential expression of Pax6 and Tbr2 is linked to differentiation from radial glia to intermediate neural progenitors^30^. Intermediate neural progenitors produce glutamatergic projection neurons for all cortical layers^31^. Therefore, riboflavin deficiency during cortical neurogenesis influences the neuronal differentiation of radial glia, but not cell proliferation, causing atrophy of cortical neurons (Fig. 6b). The molecular mechanisms underlying riboflavin-dependent neuronal differentiation will be elucidated in future studies.

Disruption of the *Slc52a3* gene affects the development of the cerebral cortex even though RFVT3 is hardly expressed in the brain. Our previous study showed that RFVT3 was highly expressed in the placenta and the placental [^3^H]riboflavin transport capacity in *Slc52a3*−/− fetuses was significantly decreased at E16.5^12^. Reduction of placental transport to *Slc52a3*−/− fetuses decreased riboflavin levels in fetal plasma and, consequently, in their tissues. In this study, excess riboflavin supplementation by subcutaneous tablet implantation and oral administration to the mother rescued the hypoplasia phenotype in developing embryos owing to passive diffusion of riboflavin through the placenta. The *SLC52A2* gene, which encodes RFVT2, is expressed in several tissues including the brain^9^, where it affects tissue distribution of riboflavin. Hence, disruption of *Slc52a3* could decrease the transport of riboflavin via the placenta to the fetus without affecting its tissue distribution.

BVVL syndrome patients with mutations in *SLC52A3* commonly present with bulbar palsy, hearing loss, muscle weakness, and respiratory symptoms in infancy or later in childhood^26,27^. Neurological diagnostic tests have shown peripheral neuropathy, anterior horn dysfunction, and chronic denervation in most cases. Although brain magnetic resonance imaging (MRI) revealed no remarkable changes, abnormal T2-weighted intensities have been sometimes described in cerebellar, cortical, subcortical (basal ganglia and external capsule), and brainstem regions of some patients with *SLC52A3* mutations^27^. Brain malformations, especially cerebral cortical hypoplasia found in *Slc52a3*−/− mice, are similar to those in the above patients. Recent neuropathological features of the central nervous system in two patients with different *SLC52A3* mutation types reflected the above clinical phenotype, although the degree of atrophy of cerebellar Purkinje cells was somewhat different between the two patients. It has been suggested that different mutations might influence the length of the disease and degree of atrophy of specific brain structures^15^. In future studies, model mice with knock-in patient mutations will clarify the relationship between the pathology and symptoms of each BVVL syndrome patient.

In conclusion, RFVT3 contributes to riboflavin homeostasis in embryos via placental transport. The *Slc52a3* gene disruption resulted in atrophy of the cerebral cortex, and this was associated with abnormal differentiation of radial glia during the embryonic period. Hence, riboflavin appears to play an important role in the embryonic development of the cerebral cortex.

## Methods

### Mice

*Slc52a3*−/− mice in a C57BL/6 background (C57BL/6N-2310046K01Rik^tm1a(KOMP)Wtsi^) were obtained from the Knockout Mouse Project Repository and were generated as described previously^12^. Mice were maintained under a 12-h light/dark cycle and provided a standard chow diet (F-2; Funabashi farm) and water ad libitum before experiments. To determine the genotype of the offspring, genomic DNA was isolated from tail biopsies, and PCR analysis was performed as previously reported^12^. Heterozygous male and female mice were mated overnight, and vaginal plugs were checked the following morning. Plug detection was considered to correspond to day 0.5 of pregnancy (E0.5). Brains were dissected at E13.5 and P0. Animal experiments were conducted in accordance with the Guidelines for Animal Experiments of Kyoto University. All protocols were approved by the Animal Research Committee, Graduate School of Medicine, Kyoto University (permission number: MedKyo20121).

### Staining of brain sections

Brain samples were collected from P0 pups and E13.5 embryos. Brains were fixed for 2 h in 4% paraformaldehyde. For hematoxylin and eosin (H&E) staining, a standard protocol for dehydration and paraffin infiltration was used^32^. Brain samples from P0 mice were cut into 5-μm sections and stained with Mayer’s Hematoxylin Solution and 1% eosin Y ethanol solution (FUJIFILM Wako Pure Chemical).

For immunostaining, brain samples were washed in phosphate-buffered saline containing 0.1% Tween 20 (PBS-T), soaked sequentially in 15% sucrose for 2 h and 30% sucrose overnight, and finally frozen in optimal cutting temperature compound (Sakura Finetek Japan). For BrdU analysis, pregnant females were dissected 1 h after the intraperitoneal injection of BrdU (100 mg/kg; Sigma) and processed for BrdU immunohistochemistry. Cryostat sections (16 μm in thickness) from the telencephalon were prepared using a Leica CM1850 cryostat. Sections were rehydrated in PBS-T, followed by antigen retrieval using 10 mM sodium citrate buffer (pH 6.0) for 10 min at 95 °C. After blocking with 5% goat serum, immunofluorescence staining with primary antibodies was performed at 4 °C overnight. Primary and secondary antibodies were diluted in 5% normal goat serum and incubated with the samples for 1 h at room temperature. Stained sections were mounted in VECTASHIED Mounting medium (H-1500; Vector Laboratories) with 4′,6-diamidino-2-phenylindole (DAPI) and scanned with a Nikon A1RMP confocal microscope. The following primary antibodies were used: rabbit anti-Cux1 (layers II–IV, 1:200; Santa Cruz Biotechnology), rabbit anti-Tbr2 (subventricular zone, 1:500; Abcam), rabbit anti-Ki67 (1:400; Abcam), mouse anti-Tuj1 (1:500; Covance), mouse anti-Pax6 (ventricular zone, 1:200; Developmental Studies Hybridoma Bank), rat anti-Ctip2 (layers V and VI, 1:200; Abcam), and rat anti-BrdU (S-phase, 1:200; Abcam). Appropriate anti-rabbit, anti-mouse, or anti-rat Highly Cross-Absorbed Secondary Antibodies conjugated with Alexa Fluor 488 or 594 (1:500; Invitrogen) were used.

The TUNEL assay was carried out on brain sections from P0 pups and E13.5 embryos using a MEBSTAIN Apoptosis TUNEL Kit (MBL) in accordance with the manufacturer’s instructions.

In situ hybridization was performed as previously described^12^. Briefly, *Slc52a3*-specific probes (position 1045–1645 in the open reading frame) were added at a concentration of 300 ng/mL in Prove Diluent-1 (Genostaff) and incubated with the samples at 60 °C for 16 h. Sections were counterstained with Kernechtrot stain solution (Muto Pure Chemicals).

### Image analysis and quantification

Coronal sections containing the dorsal telencephalon close to the diencephalon were used for quantification. The thickness of the total cortex was defined as the distance between the apical surface and basal lamina. The thickness of the intermediate zone was defined as the distance between the cortical plate and the ventricular zone. The thickness of the Cux1^+^, Ctip2^+^, Tuj1^+^, and Pax6^+^ layers and the cell numbers were measured based on confocal micrographs using NIS-Elements AR analysis software. Quantification of Tbr2^+^ and BrdU^+^ areas was performed based on confocal micrographs using ImageJ software. For each marker, the thickness of a particular area relative to the total cortex area or ventricular zone was calculated and expressed as a percentage.

### Riboflavin supplementation in mice

Implant tablets weighing 150 mg (5 mg riboflavin/tablet) were prepared by direct compression at room temperature using the AUTOTAB-500 instrument (Ichihashi Seiki). The tablets contained 3.3% riboflavin (Sigma), 16.5% microcrystalline cellulose (Avicel PH-101; Sigma), 0.2% fumed silica (Aerosil), 0.7% magnesium stearate, and 79.3% lactose (Pfizer). Placebo tablets containing 16.5% microcrystalline cellulose, 0.2% fumed silica, 0.7% magnesium stearate, and 82.6% lactose were used in the control group. *Slc52a3*+/− female mice were supplemented with a riboflavin tablet by subcutaneous implantation and were given 50 mg/L of riboflavin in drinking water ad libitum from the start of crossbreeding.

### Measurement of riboflavin, FMN, and FAD

Brain samples were collected from P0 pups. The concentrations of riboflavin, FMN, and FAD in brain samples were measured by high-performance liquid chromatography as described previously^12^.

### Real-time PCR

Real-time PCR was performed as described previously^12^. For *Slc52a3* and *Slc52a2* expression analysis, total RNA was isolated from WT mouse brains at P0 and E13.5 using the RNeasy Mini Kit (Qiagen) and then reverse transcribed. TaqMan gene expression assays for *Slc52a3* (Mm00510189_m1) and *Slc52a2* (Mm01205717_g1) were from Life Technologies. For *Tbr2* and *Pax6* analysis, total RNA was isolated from the cerebral cortex using the RNeasy Mini Kit and reverse transcribed. TaqMan gene expression assays for *Tbr2* (Mm01351985_m1) and *Pax6* (Mm00443081_m1) were from Thermo Fisher Scientific. Relative expression levels were normalized to glyceraldehyde 3-phosphate-dehydrogenase (*GAPDH*) (Mm99999915_g1).

### Statistics

Statistical analyses were performed using GraphPad Prism software (version 8.3.1). All values are expressed as the mean ± S.E.M. Differences were analyzed for significance using an unpaired Student’s *t*-test. Significance between variables is shown based on the P-value obtained (*P < 0.05, **P < 0.01, ***P < 0.001).

## Supplementary information

Supplementary Information.

## References

[1] Depeint F, Bruce WR, Shangari N, Mehta R, O'Brien PJ (2006). Mitochondrial function and toxicity: Role of the B vitamin family on mitochondrial energy metabolism. Chem. Biol. Interact..

[2] Powers HJ (2003). Riboflavin (vitamin B-2) and health. Am. J. Clin. Nutr..

[3] Thakur K, Tomar SK, Singh AK, Mandal S, Arora S (2017). Riboflavin and health: A review of recent human research. Crit. Rev. Food Sci. Nutr..

[4] Colombo B, Saraceno L, Comi G (2014). Riboflavin and migraine: The bridge over troubled mitochondria. Neurol. Sci..

[5] Ogunleye AJ, Odutuga AA (1989). The effect of riboflavin deficiency on cerebrum and cerebellum of developing rat brain. J. Nutr. Sci. Vitaminol..

[6] Yonezawa A, Inui K (2013). Novel riboflavin transporter family RFVT/SLC52: Identification, nomenclature, functional characterization and genetic diseases of RFVT/SLC52. Mol. Aspects Med..

[7] Yamamoto S (2009). Identification and functional characterization of rat riboflavin transporter 2. J. Biochem..

[8] Yonezawa A, Masuda S, Katsura T, Inui K (2008). Identification and functional characterization of a novel human and rat riboflavin transporter, RFT1. Am. J. Physiol. Cell Physiol..

[9] Yao Y (2010). Identification and comparative functional characterization of a new human riboflavin transporter hRFT3 expressed in the brain. J. Nutr..

[10] Yoshimatsu H (2014). Functional involvement of RFVT3/SLC52A3 in intestinal riboflavin absorption. Am. J. Physiol. Gastrointest. Liver Physiol..

[11] Jin C (2017). Riboflavin transporters RFVT/SLC52A mediate translocation of riboflavin, rather than FMN or FAD, across plasma membrane. Biol. Pharm. Bull..

[12] Yoshimatsu H (2016). Disruption of Slc52a3 gene causes neonatal lethality with riboflavin deficiency in mice. Sci. Rep..

[13] Green P (2010). Brown-Vialetto-Van Laere syndrome, a ponto-bulbar palsy with deafness, is caused by mutations in c20orf54. Am. J. Hum. Genet..

[14] Khadilkar SV, Faldu HD, Udani V, Patil SB, Malvadkar S (2017). Reversible posterior column dysfunction in Brown-Vialetto-Von Laere syndrome. Muscle Nerve.

[15] Manole A (2017). Clinical, pathological and functional characterization of riboflavin-responsive neuropathy. Brain.

[16] Bosch AM (2011). Brown-Vialetto-Van Laere and Fazio Londe syndrome is associated with a riboflavin transporter defect mimicking mild MADD: A new inborn error of metabolism with potential treatment. J. Inherit. Metab. Dis..

[17] Anand G (2012). Early use of high-dose riboflavin in a case of Brown-Vialetto-Van Laere syndrome. Dev. Med. Child Neurol..

[18] Udhayabanu T (2016). SLC52A2 [p.P141T] and SLC52A3 [p.N21S] causing Brown-Vialetto-Van Laere Syndrome in an Indian patient: First genetically proven case with mutations in two riboflavin transporters. Clin. Chim. Acta.

[19] Hossain MA (2017). Early onset of Fazio-Londe syndrome: The first case report from the Arabian Peninsula. Hum. Genome Var..

[20] Bashford JA, Chowdhury FA, Shaw CE (2017). Remarkable motor recovery after riboflavin therapy in adult-onset Brown-Vialetto-Van Laere syndrome. Pract. Neurol..

[21] Thulasi V, Veerapandiyan A, Pletcher BA, Tong CM, Ming X (2017). A case of Brown-Vialetto-Van Laere syndrome due to a novel mutation in *SLC52A3* gene: Clinical course and response to riboflavin. Child Neurol. Open.

[22] Camargos S, Guerreiro R, Bras J, Mageste LS (2018). Late-onset and acute presentation of Brown-Vialetto-Van Laere syndrome in a Brazilian family. Neurol. Genet..

[23] Chaya S (2018). The first case of riboflavin transporter deficiency in sub-Saharan Africa. Semin. Pediatr. Neurol..

[24] Gowda VK, Udhayabanu T, Varalakshmi P, Srinivasan VM, Ashokkumar B (2018). Fazio-Londe syndrome in siblings from India with different phenotypes. Brain Dev..

[25] Woodcock IR (2018). Genetic, radiologic, and clinical variability in Brown-Vialetto-van Laere syndrome. Semin Pediatr. Neurol..

[26] Jaeger B, Bosch AM (2016). Clinical presentation and outcome of riboflavin transporter deficiency: Mini review after five years of experience. J. Inherit. Metab. Dis..

[27] O'Callaghan B, Bosch AM, Houlden H (2019). An update on the genetics, clinical presentation, and pathomechanisms of human riboflavin transporter deficiency. J. Inherit. Metab. Dis..

[28] Rizzo F (2017). Genome-wide RNA-seq of iPSC-derived motor neurons indicates selective cytoskeletal perturbation in Brown-Vialetto disease that is partially rescued by riboflavin. Sci. Rep..

[29] Greig LC, Woodworth MB, Galazo MJ, Padmanabhan H, Macklis JD (2013). Molecular logic of neocortical projection neuron specification, development and diversity. Nat. Rev. Neurosci..

[30] Englund C (2005). Pax6, Tbr2, and Tbr1 are expressed sequentially by radial glia, intermediate progenitor cells, and postmitotic neurons in developing neocortex. J. Neurosci..

[31] Hevner RF (2019). Intermediate progenitors and Tbr2 in cortical development. J. Anat..

[32] Feldman AT, Wolfe D (2014). Tissue processing and hematoxylin and eosin staining. Methods Mol. Biol..

