# Supplementary material for: Effect of riboflavin deficiency on development of the cerebral cortex in Slc52a3 knockout mice
Source: Sci Rep. 2020 Oct 28;10:18443. doi: 10.1038/s41598-020-75601-9 (PMC7595085; doi:10.1038/s41598-020-75601-9)
Supplement: Supplementary file 1 — Supplementary Information. [file 41598_2020_75601_MOESM1_ESM.docx]

**SUPPLEMENTARY INFORMATION**

**Effect of riboflavin deficiency on development of the cerebral cortex in *Slc52a3* knockout mice**

Congyun Jin^1,2^, Atsushi Yonezawa^1,2*^, Hiroki Yoshimatsu^1,2^, Satoshi Imai^1^, Madoka Koyanagi^1,2^, Kaori Yamanishi^1,2^, Shunsaku Nakagawa^1^, Kotaro Itohara^1^, Tomohiro Omura^1^, Takayuki Nakagawa^1^, Junya Nagai^3^, Kazuo Matsubara^1^

^1^Department of Clinical Pharmacology and Therapeutics, Kyoto University Hospital, 54 Shogoin Kawahara-cho, Sakyo-ku, Kyoto 606-8507, Japan

^2^Graduate School of Pharmaceutical Sciences, Kyoto University, 54 Shogoin Kawahara-cho, Sakyo-ku, Kyoto 606-8507, Japan

^3^Department of Pharmaceutics, Osaka University of Pharmaceutical Sciences, 4-20-1 Nasahara, Takatsuki, Osaka 569-1094, Japan

*Corresponding author: Atsushi Yonezawa, Ph.D.

Department of Clinical Pharmacology and Therapeutics, Kyoto University Hospital, 54 Shogoin Kawahara-cho, Sakyo-Ku, Kyoto 606-8507, Japan

TEL number: +81-75-751-3582

FAX number: +81-75-751-4207

E-mail: ayone@kuhp.kyoto-u.ac.jp

**
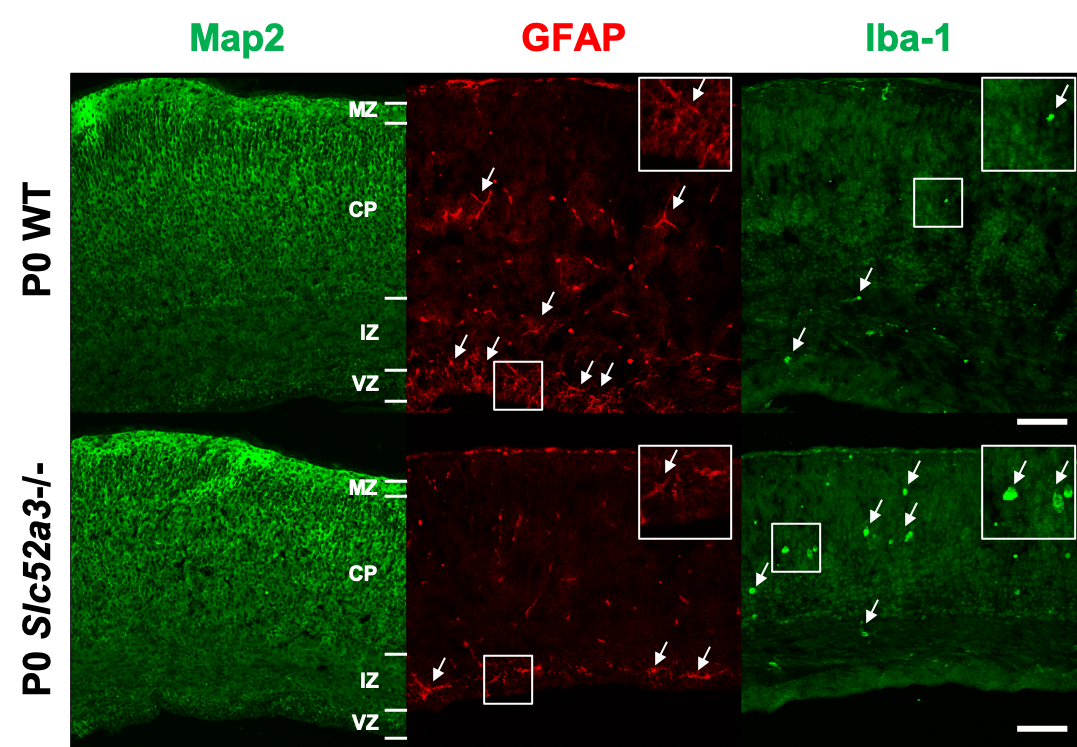
**

**Supplementary Figure 1. Immunostaining of neurons, astrocytes, and microglia in the cerebral cortex at P0 in WT and *Slc52a3*-/- mice.**

Confocal micrographs of coronal cortex sections from the indicated genotypes at P0 stained with antibodies against Map2 (green), GFAP (red), and Iba-1 (green). Arrows show positive staining for GFAP and Iba-1. Insets show magnified views of the boxed regions. Scale bars, 100 μm. MZ, marginal zone; CP, cortical plate; IZ, intermediate zone; VZ, ventricular zone.

**
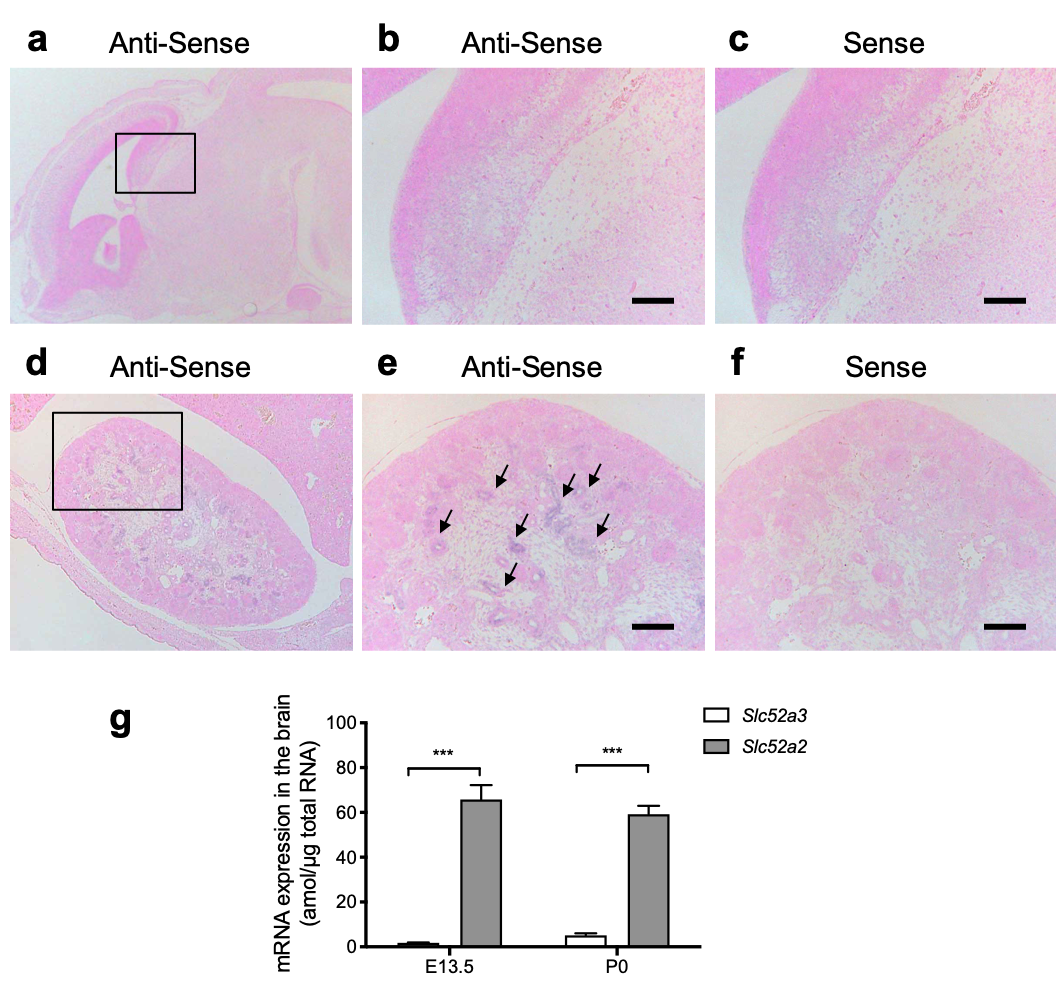
**

**Supplementary Figure 2. Distribution of riboflavin transporters in the mouse brain.**

(a) *In situ* hybridization showing mRNA expression of *Slc52a3* in the brain at E16.5. The boxed area indicates the magnified region in panels b and c. (b) Solid flames in the hippocampus. (c) Negative control showing hybridization with a sense probe for *Slc52a3*. (d–f) Positive control showing mRNA expression of *Slc52a3* in the kidney. Arrows (blue signal) show positive staining for *Slc52a3*. Scale bars, 100 μm. (g) Real-time PCR results showing mRNA expression of *Slc52a3* and *Slc52a2* in WT mouse brains at P0 (n = 4 from 3 dams) and E13.5 (n = 6 from 2 dams). Each bar represents the mean ± S.E.M., *** P < 0.001, E13.5 vs P0.

**
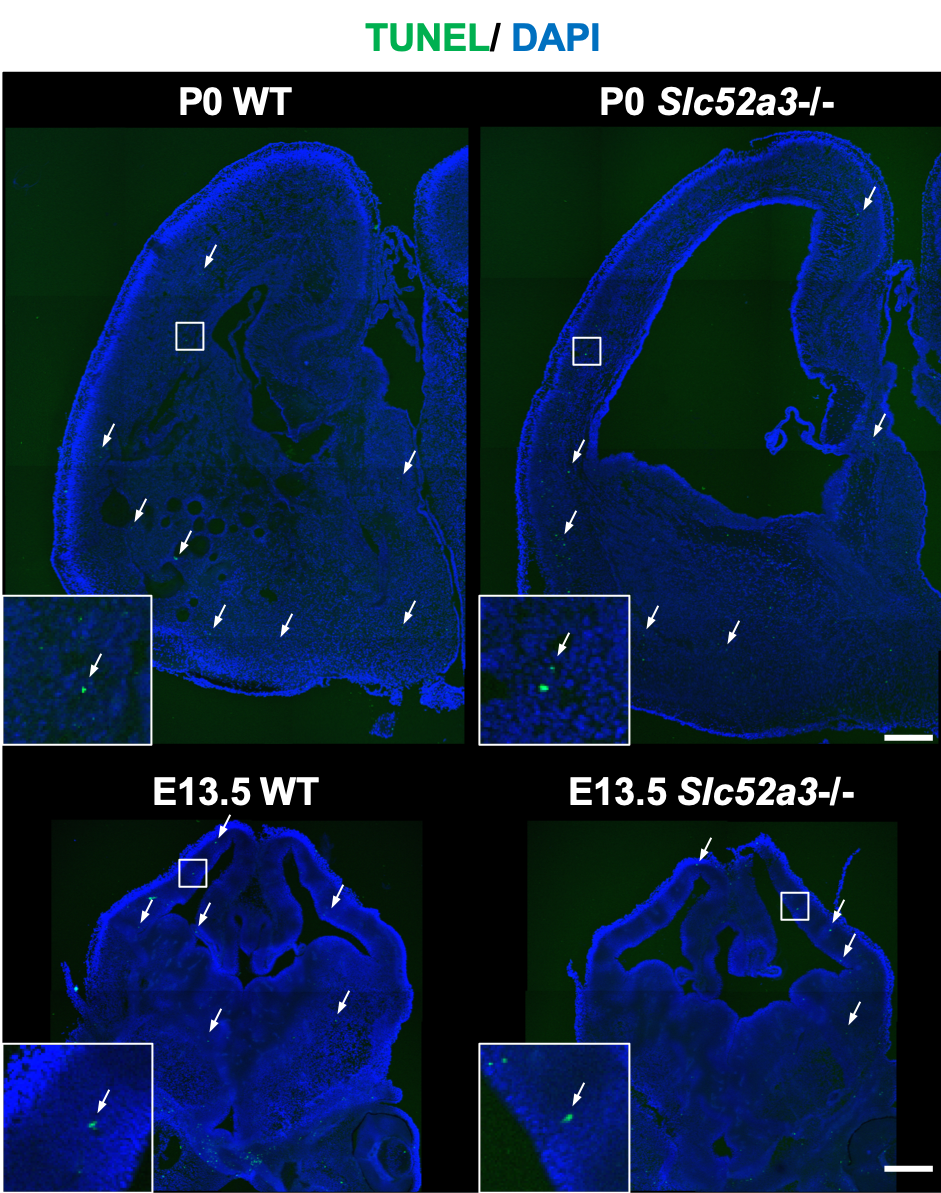
**

**Supplementary Figure 3. Analysis of apoptosis in the developing brain of WT and *Slc52a3*-/- mice.**

TUNEL assay on brain sections from P0 pups and E13.5 embryos of each genotype. Arrows show positive TUNEL staining (green signal). Insets show magnified views of the boxed regions. Scale bars, 200 μm.

**
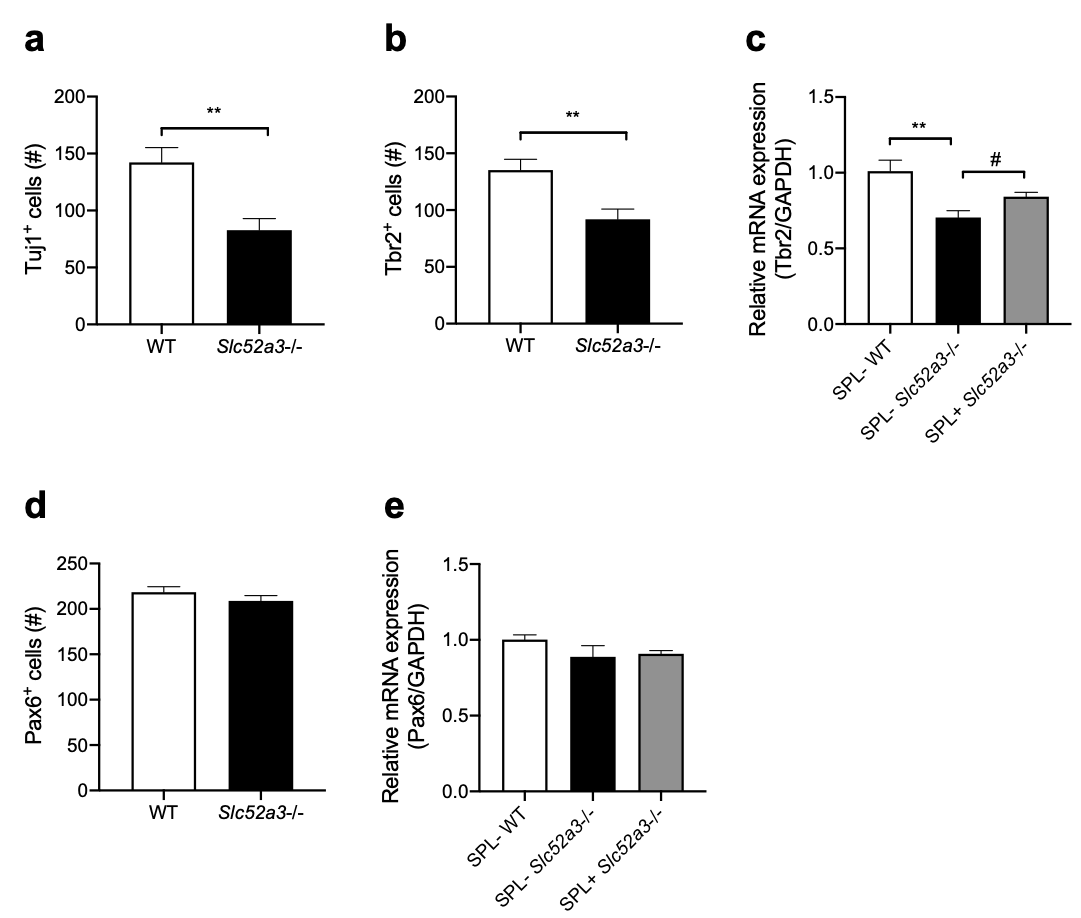
**

**Supplementary Figure 4. Tbr2 and Pax6 cell numbers and expression levels in the cerebral cortex of WT and *Slc52a3*-/- mice with (SPL+) or without (SPL-) riboflavin supplementation.**

Quantification of the number of Tuj1^+^ (a), Tbr2^+^ (b), and Pax6^+^ (d) cells per 85-μm column (n = 11 from 9 dams). Relative mRNA expression levels of Tbr2 (c) and Pax6 (e) in SPL- WT (n = 5 from 3 dams), SPL- *Slc52a3-/-* (n = 5 from 3 dams), and SPL+ *Slc52a3-/-* (n = 9 from 3 dams) mice obtained by RT-PCR. Expression levels were normalized to *GAPDH*. Each bar represents the mean ± S.E.M., ** P < 0.01, SPL- WT vs SPL- *Slc52a3*-/-; ^#^ P < 0.05, SPL- *Slc52a3*-/- vs SPL+ *Slc52a3*-/-.
